# The pursuit of polymethine fluorophores with NIR-II emission and high brightness for *in vivo* applications

**DOI:** 10.1039/d2sc03136a

**Published:** 2022-08-27

**Authors:** Xuan Zhao, Fan Zhang, Zuhai Lei

**Affiliations:** Minhang Hospital and Key Laboratory of Smart Drug Delivery, Ministry of Education, School of Pharmacy, Fudan University Shanghai 201203 China lei_zuhai@fudan.edu.cn; Department of Chemistry, State Key Laboratory of Molecular Engineering of Polymers, Shanghai Key Laboratory of Molecular Catalysis and Innovative Materials and iChem, Fudan University Shanghai 200433 China

## Abstract

Polymethine cyanine dyes, as the most important class of organic near-infrared-II (NIR-II) fluorophores, recently received increasing attention due to their high molar extinction coefficients, intensive fluorescence brightness, and flexible wavelength tunability for fluorescent bioimaging applications. Very recently, remarkable advances have been made in the development of NIR-II polymethine fluorophores with improved optical performance, mainly including tunable fluorescence, improved brightness, improved water solubility and stability. In this review, we summarize the recent research advances in molecular tailoring design strategies of NIR-II polymethine fluorophores, and then emphasize the representative bioimaging and biosensing applications. The potential challenges and perspectives of NIR-II polymethine fluorophores in this emerging field are also discussed. This review may provide guidance and reference for further development of high-performance NIR-II polymethine fluorophores to boost their clinical translation in the future.

## Abstract 2

Overview of historical development for polymethine fluorophores with NIR-II emission and high brightness for *in vivo* applications.

## Graphical abstract

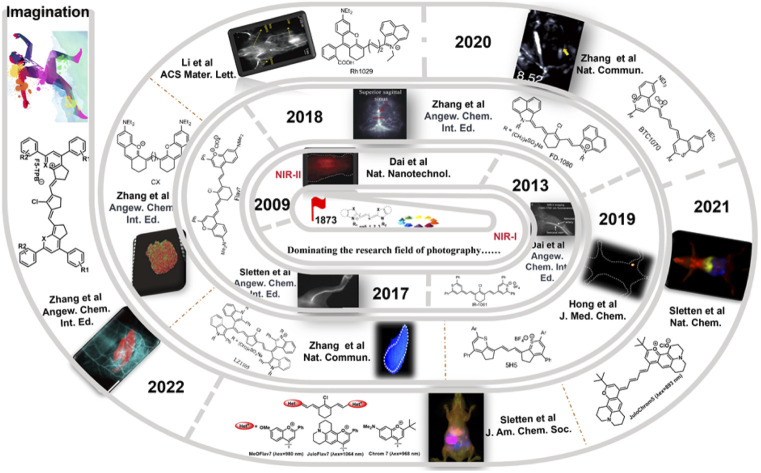

## 1 Introduction

Fluorescence imaging, as a non-invasive or minimally invasive diagnostic imaging modality, holds great promise for improving disease detection and image-guided surgery in preclinical studies and clinical trials.^1,2^ The fluorescence imaging technique has multiple competitive advantages, mainly including unprecedented spatiotemporal resolution, high selectivity and high sensitivity, *in situ* real-time detection, non-invasiveness, safety, and low cost.^3,4^ Such a simple but highly sensitive imaging technique allows for profoundly exploring the physiological and pathological processes at molecular, cellular, and tissue level in real-time and in multiple dimensions.^5,6^ To date, fluorescence imaging has been extensively employed for various biosensing and bioimaging applications, including but not limited to immunofluorescence assays, super-resolution imaging, 3D imaging, and image-guided clinical surgery, *etc.*^1^ And now, indocyanine green (ICG: *λ*_ex_ = 808 nm, *λ*_em_ = 822 nm) and methylene blue (MB: *λ*_ex_ = 665 nm, *λ*_em_ = 686 nm) have been successfully developed and approved by the US Food and Drug Administration (FDA) for clinical use in humans, such as angiography, sentinel lymph node biopsy (SLNB), and fluorescence navigation surgery.^7,8^ Compared with visible spectral fluorescence (VIS: 400–650 nm), NIR-I (650–900 nm) fluorescence imaging can improve tissue penetration depth and reduce background interference to some extent due to reduced photon absorption and scattering, and less autofluorescence. Over the past few decades, NIR-I fluorescence imaging has undergone explosive growth, but NIR-I technique still suffers from the roadblock of low photon tissue penetration depth which severely restricts its further *in vivo* biological applications. Hence, it remains a great challenge to meet the multilevel and diverse requirements for high-definition imaging results through NIR-I fluorescence imaging. To this end, researchers never stop to pursue the more high-performance fluorophores to expand their *in vivo* biological applications.

Since 2009, fluorescence imaging in the second near-infrared (NIR-II, 1000–1700 nm) window, as a cutting-edge imaging technique, has received considerable attention worldwide due to its low photon scattering, minimal autofluorescence, and deep tissue penetration depth.^9–11^ The NIR-II fluorescence imaging technique can markedly improve the sensitivity and specificity of disease detection and diagnosis in biomedical research and clinical practice. More recently, remarkable achievements have been made *in vivo* fluorescence imaging with NIR-II emission.^12–14^ And a range of NIR-II molecular fluorophores have been successfully developed and employed for diverse biosensing and bioimaging applications. Though the research on NIR-II fluorescent imaging is continuously flourishing, currently available high-performance NIR-II fluorophores remain extremely scarce which largely limits the further development of NIR-II imaging technology. Currently, most of the reported fluorescence quantum yields of NIR-II fluorophores remain far lower than that of NIR-I fluorophores on account of the small HOMO–LUMO gap and poor structural rigidity, which generally leads to weak fluorescent brightness in a living system.^15^ During the development of molecular and structural engineering, most NIR-II fluorophores suffer from the pending dilemma that wavelength and brightness can't be firmly balanced, namely long enough wavelength with low brightness or high brightness with short wavelength.^16^ Furthermore, poor water solubility and low stability also have limited their further applications and clinical transformation for NIR-II imaging techniques. Therefore, intensive effort should be continually made to develop NIR-II fluorophores with a longer wavelength in the NIR-II window, high brightness, favorable stability, and good biocompatibility. Until now, small-molecular NIR-II fluorophores primarily include these three structural types: donor–acceptor–donor (D–A–D), polymethine (D–π–A), and BODIPY scaffolds.^17–19^ Among them, polymethine fluorophores, as a class of indispensable NIR-II fluorophores for chemical and biological research, received remarkable attention due to their high molar extinction coefficients, intensive brightness, and flexile wavelength tunability for versatile biomedical applications.^5,20,21^ And polymethine fluorophores have been successfully explored to enable the acquisition of high-fidelity imaging at wavelengths beyond 1000 nm, including commercially available IR-1048 (ref. 22) and IR-1061,^23^ flavylium polymethine dye (Flav7),^24^ Flav7 analog dyes,^25^ and FD-1080,^26^*etc.* However, there are still some unresolved critical issues in the development of NIR-II polymethine fluorophores for further applications and clinical trials due to unsatisfactory fluorescent brightness, poor molecular stability, and bad biocompatibility.

In this review, we systemically summarize recent research advances in the development of NIR-II polymethine fluorophores for biosensing and bioimaging in living systems. We emphatically introduce their rational productive manipulation strategy, spectroscopic properties, and highlight some selected biological applications. Meanwhile, we also discuss the challenges and prospects in the future development of polymethine fluorophores in this newly emerging field.

## 2 Productive manipulation of polymethine fluorophores

Since 1856, cyanine dyes have almost dominated the research field and applications of photography and other relevant biological and medicinal research fields.^27^ Generally, the classic cyanine dyes are composed of two nitrogen-containing heterocycles, one of which is positively charged and is covalently linked by a tunable conjugated chain of an odd number of carbon atoms to the other nitrogen center.^28^ This unique molecular structure imparts cyanine dyes with multiple intrinsically inherent advantages, including flexible synthesis and modification, and wavelength-tunable absorption and emission. In the past few decades ago, there were already many polymethine fluorophores with a long-wavelength absorption or emission, but not for further applications due to the limitation of supporting imaging instruments and systems.^29^ With the development of NIR-II fluorescence imaging system, polymethine cyanine dyes received increasing attention for wide-ranging biomedical applications due to their above-mentioned multiple inherent advantages. Polymethine dye scaffolds are popular and promising candidates for constructing NIR-II fluorescent contrast agents. The length of the polymethine chain, structures of heterocycle, nature and position of substituents, electronic asymmetry, interactions of fluorophores, and structures of ion-pairs can influence the spectroscopic properties of polymethine cyanine dyes.^30^ Among them, the polymethine chain is the major structural factor determining the spectroscopic properties of polymethine fluorophores. Lengthening the polymethine chain can effectively extend the π-conjugate system promoting the emission wavelength bathochromic shift of polymethine fluorophores, even beyond 1000 nm. But polymethine cyanine dyes with more than seven methines, namely beyond Cy7, are well-known to suffer from the bottleneck of “cyanine limit” and undergo a symmetry-breaking Peierls transition process which results in an unsymmetric distribution of π-electron density in the ground state, localized positive charge, and substantial bond length transformation along with the π-conjugation chain.^31^ Compared with symmetrical counterparts, the polar unsymmetric cyanine fluorophores exhibit a broader charge-transfer type absorption and lower fluorescence quantum yields. Since the Peierls transition is vigorously favored in aqueous solution, the “cyanine limit” problem is a non-negligible main holdback for small-molecular polymethine dyes design that largely limits the preparation of high-performance NIR-II polymethine fluorophores for diverse biosensing and bioimaging applications. Otherwise, the different structures of heterocycle also have an extremely significant influence on the spectroscopic properties of polymethine fluorophores. In this way, it is possible to obtain long-wavelength polymethine fluorophores through rationally modifying heterocycle, but not need extending the polymethine chain length. Such an effective strategy allows to obtain more stable polymethine fluorophores than cyanine dyes with long polymethine chain. Introducing substituents with various electronic characteristics into different positions of the heteroradicals can also regulate the extent of vibrational interactions in polymethine dye molecules and then influence their spectroscopic properties. It is also a good option to fine-tune the optical performance of polymethine fluorophores by rationally introducing selective substituents. To enable real-time and high-contrast bioimaging and biosensing, an ideal NIR-II polymethine fluorophore should comply with three crucial principles: (1) long absorption/emission wavelengths in the NIR-II window; (2) high NIR-II fluorescent brightness in biological system; (3) enhanced fluorophore chemostability and photostability.

**Fig. 1:**
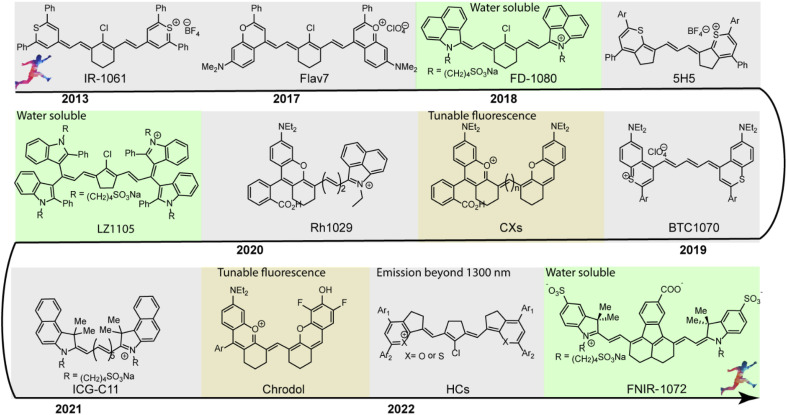
Typical chemical structures of NIR-II polymethine fluorophores applied for NIR-II bioimaging and biosensing are listed in the timeline.

In the past decade, considering the significant competitive advantages of NIR-II imaging, intensive efforts have been devoted to designing and synthesizing NIR-II polymethine fluorophores with improved optical properties, mainly including longer wavelength, higher brightness, better biocompatibility, and superior optical/physiological stability. In this section, we will detailly discuss the molecular tailoring strategies for NIR-II polymethine fluorophores based on these above-mentioned pivotal factors to avoid the “cyanine limit” problem. The typical chemical structures of NIR-II polymethine dyes recently applied for living system applications are summarized in Fig. 1. The corresponding photophysical data and bioimaging applications are summarized in Table 1.

**Table 1:** Photophysical data and biological imaging applications of representative NIR-II polymethine fluorophores

Cmpd	Fluorophore	Solvent	*λ*_abs_/*λ*_em_ [nm]	*Φ*_NIR-II_ [%]	*ε* [M^−1^ cm^−1^]	*ε* × *Φ*_NIR II_ [M^−1^ cm^−1^]	Water-soluble	Applications	Ref.
1	BTC1070	DCM	1014/1070	0.09^a^	115 000	104	By micelles	Ratiometric fluorescence quantification of gastric pH	32
2	Flav7	DCM	1026/1045	0.53^a^	23 600	125	By micelles	Vasculature imaging	24
3	Rh1029	DCM	1029/1093	0.33^b^	19 000	62.7	By micelles	Vasculature imaging	33
4	LZ1105	DMSO	1058/1100	3.89^a^	148 000	5757.2	Yes	Dynamic vascular imaging	34
5	FD1080	FBS	1046/1080	5.94^a^	29 672	1763	Yes	Hindlimb vasculature and brain vessel bioimaging	26
6	IR1061	DCM	1061/1100	0.59^c^	238 000	1400	By micelles	Vascular imaging	23
7	5H5	AcN	1069/1125	2.6^a^	34 200	889	PEGylated	Blood vessel and tumor imaging	35
8	FNIR-1072	MeOH	1072/1103	0.13^a^	120 000	156	Yes	Targeted multicolor *in vivo* imaging	36
9	Cy-PA	DCE	1087/1120	0.09^a^	49 800	44.82	By micelles	Vasculature imaging	37
10	CX-3	CHCl_3_	1089/1140	0.091^a^	104 712	95.3	By micelles	Ratiometric fluorescence detection of hepatotoxicity	38
11	HC1222	DCE	1180/1222	0.016^a^	117 320	18.8	By micelles	Dual-color imaging of the circulatory system and metabolic organs	39
12	HC1342	DCE	1286/1342	0.015^a^	108 410	16.3	By micelles	Dual-color imaging of the circulatory system and metabolic organs	39

a The relative fluorescence quantum yield by using IR-26 in 1,2-dichloroethane as the reference (*Φ*_FL_ = 0.05%).

b Using IR1061 in dichloroethane as the reference (*Φ*_FL_ = 0.53%).

c Absolute quantum yield.

### 2.1 Tunable absorption and emission in the NIR-II window

**Fig. 2:**
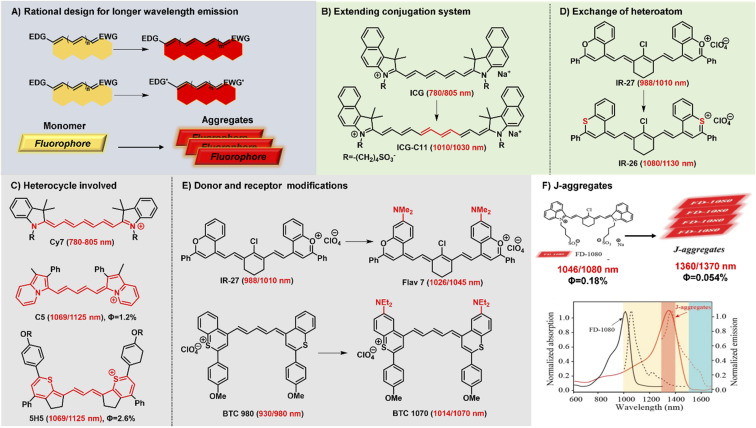
Schematic diagram of structure tailoring strategies for longer-wavelength polymethine fluorophores. (A) Representative design strategies for longer emission wavelengths; (B) extending the conjugation chain; (C) heterocycle involved; (D) exchanging of heteroatoms; (E) donor and accept modifications; (F) constructing fluorophore J-aggregates. Reproduced with permission from ref. 47. Copyright 2021 American Chemical Society.

Polymethine fluorophores have conjugated π-electrons and a push–pull structural element. And the intramolecular charge transfer (ICT) usually occurs in push–pull molecular π-conjugated systems containing an electron donor (D) and an electron acceptor (A). Generally, NIR-II fluorophores display a relatively smaller HOMO–LUMO gap than NIR-I chromophores. Consequently, through reasonably regulating the charge transfer effect between different electron donors and acceptors to achieve a smaller HOMO–LUMO gap, the fluorescence emission of polymethine fluorophores can be extended to the NIR-II spectrum range. The rational and effective design strategies have been explored to develop polymethine fluorophores with NIR-II emission wavelength, mainly including these three types: (a) extending conjugation of π-electrons, (b) increasing the electron density of the donor or/and decreasing the electron density of the acceptor, (c) constructing fluorescent J-aggregates for larger red-shifted absorption and emission (Fig. 2A).^16^

Lengthening the polymethine chain is a classic and valid strategy to push the wavelength redshift, but this strategy can simultaneously induce the negative result of compromised fluorescence quantum yield, decreased stability, and the loss of symmetric electron delocalization due to the “cyanine limit” problem. Different from extending conjugation by increasing the length of the polymethine bridge, Delcamp and Hong developed a new strategy to force all the orbitals of the indolizine and thiopyrylium moieties to participate in the resonance enlarging the conjugation system (Fig. 2C).^35,40^ Surprisingly, dyes C5 and 5H5, as Cy5 and Cy3 derivatives, respectively, can effectively absorb light and emit fluorescence in the NIR-II region whose absorption and emission wavelengths realize a more obvious bathochromic shift than Cy7 systems. In addition, Martin groups also reported two new classes of persulfonated indocyanine dyes FIN-872 and FNIR-1072 through catechol-ring and aryl-ring fusion, respectively.^36^ Compared to the catechol fusion, the different manner of aryl rings on the nonamethine scaffold can bring about the intriguing results of the maximum absorption wavelength red-shifted from 872 to 1072 nm through extending the conjugation system.

Heterocycle modification is an alternative and useful approach to construct NIR-II emissive stable polymethine fluorophores. Recent studies show that replacing the heteroatom from oxygen with a carbon or chalcogen atom can lead to a bathochromic shift.^41,42^ For example, the oxygen atoms of IR-27 were substituted by sulfur atoms, leading to an about 100 nm red-shifted absorption from 988 to 1080 nm (Fig. 2D). But this heterocycle exchange generally induces the depressing results of compromised fluorescence quantum yield and decreased stability due to heavy atom effects. Maybe it is a difficult option to firmly balance the trade-off between the wavelength and fluorescence quantum yield. Extending heterocycle conjugation or introducing electron-donating groups has been shown to bathochromically shift the polymethine fluorophores.^43^ Inspired by the IR-26 structure, Sletten's groups chose to blue-shift the absorbance to improve fluorescence quantum yield by replacing the sulfur heteroatom with oxygen in thiaflavylium (Fig. 2E).^24^ To obtain red-shifted polymethine dyes, the electron-donating dimethylamino group was introduced to compensate the conjugated system in flavylium heterocycles. Among these synthesized polymethine dyes, Flav7 has the maximal absorption and emission in the NIR-II window which is bathochromically-shifted from classic cyanine dyes by approximately 200 nm. At the same time, they also found that substituent steric effects would observably influence the photophysical properties of flavylium heptamethine dyes by regulating the degree of conjugation, so the emission wavelength of Flav7 realized a bathochromical shift of approximately 70 nm through changing the position of substituents.^44^ Otherwise, they further developed an array of tunable NIR-II emissive flavylium polymethine fluorophores by combining flavylium heterocycle with the relevant bis(phenylimine) and created a clear relation between structure and optical performance for this kind of bright NIR-II fluorescent imaging reagents.^25^ Similarly, BTC1070 bearing two electron-donating diethylamino moieties has an approximately 100 nm emission wavelength bathochromic shift compared to BTC980, which could be attributed to the strong intramolecular charge transfer effect of nitrogen atoms.^32^ Based on extending heterocycle conjugation of IR1048, Yang groups successfully designed and developed a novel heptamethine cyanine dye Cy-PA with the maximal fluorescence emission at approximately 1120 nm by introducing indolium-derived polycyclic aromatic hydrocarbons on the terminal ends of the conjugated polyene backbone.^37^

The self-aggregation behavior of fluorophores in aqueous solution is a common phenomenon in dye chemistry due to the existence of strong intermolecular van der Waals forces between these fluorophore molecules.^28^ Specially, the π-extension on polymethine chain can inevitably accelerate the self-aggregation of fluorophore molecules. Every coin has two sides, as the old saying goes. The fluorophores with a planar conjugation structure in water solution may readily orient in a head-to-tail fashion to form J-aggregates. Compared with the monomer molecules, J-aggregates, as highly ordered assembled organic dyes *via* the self-assembly processes, display the spectroscopic properties of bathochromically-shifted absorption and emission, small Stokes shift, and increased absorption coefficients.^45,46^ The unique optical properties of J-aggregates are desired and valuable for *in vivo* bioimaging applications. Therefore, it is also an attractive and efficient strategy to achieve longer wavelength fluorescent contrast agents by constructing fluorescent J-aggregates. For example, the self-assembly of amphiphilic cyanine dye FD-1080 and 1,2-dimyristoyl-*sn-glycero*-3-phosphocholine (DMPC) presents a stabilized FD-1080 J-aggregate with remarkably bathochromically-shifted absorption at 1360 nm and emission at 1370 nm (Fig. 2F).^47^ The FD-1080 J-aggregates can be utilized as imaging contrasts to afford more distinct imaging results for vasculature visualization than FD-1080 monomers. Besides, Sletten groups loaded the NIR polymethine dye IR-140 inside PEGylated hollow mesoporous silica nanoparticles to prepare stable shortwave infrared-emissive J-aggregates which enabled the *in vivo* imaging under 980 nm laser excitation with high resolution.^48^

### 2.2 Strategies to boost NIR-II fluorescent brightness

**Fig. 3:**
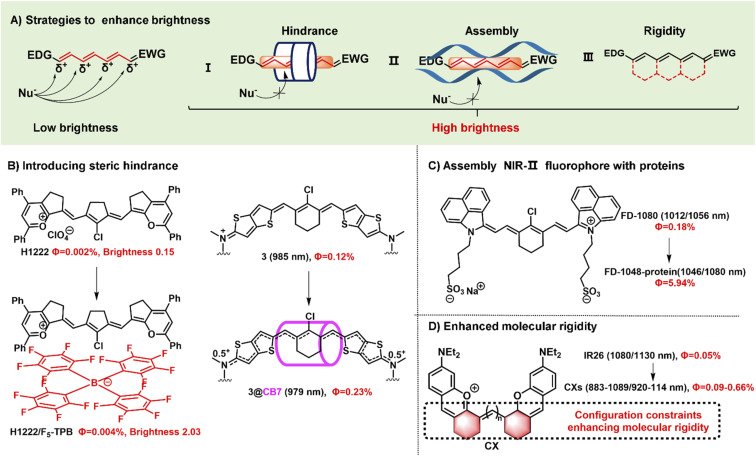
Schematic illustration of the strategies to improve fluorescent brightness of NIR- II polymethine fluorophores. (A) Scheme of the effective strategies to improve the brightness for polymethine fluorophores including (i) introducing large steric hindrance, (ii) self-assembling with protein, and (iii) improving the molecular rigidity. (B) Introducing large steric hindrance. Reproduced with permission from ref. 39. Copyright 2022 Wiley-VCH and ref. 55. Copyright 2022 American Chemical Society. (C) Assembly of NIR-II polymethine fluorophores and protein for improved optical performance. Reproduced with permission from ref. 26. Copyright 2018 Wiley-VCH. (D) enhancing the molecular rigidity. Reproduced with permission from ref. 38. Copyright 2020 Wiley-VCH.

Fluorophore brightness strongly depends on both absorption and emission properties according to the definition [brightness = coefficient (*ε*) × quantum yield (*Φ*_F_)]. An ideal fluorophore should have intense absorption and emission properties upon light excitation. Most small-molecular NIR-II polymethine fluorophores have the common characteristic of a highly bulky conjugated system with a small HOMO/LUMO energy gap which leads to the low quantum yield. In general, the fluorescent brightness of most NIR-II fluorophores (below 10^3^ M^−1^ cm^−1^) is almost two orders of magnitude lower than that of visible and NIR-I fluorophores (around 10^5^ M^−1^ cm^−1^).^49^ The weak brightness of NIR-II fluorophores in an aqueous solution is mainly attributed to their interactions with surrounding water molecules, which causes the energy loss of the excited fluorophore.^50^ To increase the quantum yield of polymethine fluorophores, some enlightening and attractive strategies have been proposed to reduce nonradiative processes by interactions with biomolecules, introduce conformational restraint on the polymethine skeleton structure, or decrease intersystem crossing by changing heavy atoms.^51,52^ Furthermore, intensive efforts to improve the brightness of polymethine dyes can take steps to reduce aggregation effects as far as possible to keep them in a molecular monomer form for effective absorbing and emitting capacity (Fig. 3A).^53^

Aggregation-caused quenching, as a common phenomenon in a biological system, is one of the leading reasons to cause the low fluorescent brightness of NIR-II polymethine fluorophores. The π-extension of polymethine chain and/or terminal heterocycle moieties is extremely necessary for absorption and emission wavelength redshift which would inevitably accelerate molecular self-aggregation through π-stacking and solvatochromism on account of the loss of symmetry, leading to broader absorption spectra, and compromised fluorescent brightness in aqueous solution.^54^ Therefore, introducing a larger steric hindrance group is a practical strategy to boost the brightness of fluorophores. For example, Zhang's group designed and developed a range of heptamethine cyanine (HC) dyes with tunable maximal absorption/emission ranging from 1100 to 1400 nm through the molecular engineering strategy (Fig. 3B).^39^ As a proof of concept, among these dyes, HC1222 and HC1342 displayed approximately 14-fold and 13-fold enhancements of fluorescent brightness in aqueous solution after counterion exchange with bulky tetrakis(pentafluorophenyl) borate (F5-TPB) reference to their parent forms. This work provides a successful paradigm to overcome the problem of fluorophore self-aggregation and fluorescence self-quenching and reserve the NIR-II multiplexed imaging capacity for high-contrast dual-color bioimaging. Besides, supermolecule is also a good strategy to introduce steric hindrance. To mitigate the “cyanine limit” and improve fluorescent brightness, Bradley groups encapsulated the newly developed cationic cyanine dye 3 with cucurbit[7]uril (CB7) through the host/guest self-assembly strategy, transforming the π-electron distribution of the cyanine fluorophore (Fig. 3B).^55^ In an aqueous solution, the dye 3 displays a strong solvatochromism phenomenon owing to the symmetry breaking of Peierls transition, leading to a broad and weak absorption and decreased fluorescence quantum yield. After the dye 3 is packaged by CB7, the surrounding CB7 primarily locates in the orientation of the fluorophore's central chlorocyclohexenyl ring, and the fluorophores reserve a symmetrical π-electron state that exhibits a sharp and intense absorption peak and 12-fold enhancement of fluorescent brightness. Therefore, the self-assembly strategy of the dye 3 and CB7 can remarkably stabilize the dye's nonpolar, unsymmetric π-electron in the ground state, and significantly enhance the fluorescent brightness. This work demonstrates that the host/guest assembly strategy can be utilized to mitigate the “cyanine limit” problem and improve the fluorescent brightness of fluorophores in the biological environment. Otherwise, Chan's group also designed and synthesized a series of polymethine-based semiconducting polymers with absorption and emission maxima at NIR-II window for bioimaging.^56^ These prepared semiconducting polymers are imparted anti-aggregation-caused quenching properties owing to the bulky architecture and have fluorescence quantum yields of 0.05–0.18% in aqueous solutions.

Packing the polymethine fluorophores with protein to form a stable protein-fluorophore complex can restrict the intramolecular rotation of fluorophores which provides an efficient approach to increase their fluorescent brightness.^54^ For example, Chen groups developed a class of biocompatible dye–protein complex as optical nanoprobes with superbright fluorescence.^58^ As compared with the dye monomer molecules, apoferritin-dye-NPs possess hundreds of times larger molar extinction coefficient, higher absolute luminescence quantum yield (up to 45-fold), and enhanced fluorescence brightness up to 2778-fold. Similarly, FD-1080 containing two sulfonic acid groups and cyclohexene group has favorable water solubility and stability with both maximal absorption and emission in the NIR-II window.^26^ Through combining with fetal bovine serum (FBS), the quantum yield of FD-1080-FBS complex can effectively increase from 0.31% to 5.94% (Fig. 3C). This strategy can remarkably improve the fluorescence quantum yield which is beneficial for bright fluorescent bioimaging *in vivo*.

Another approach to boost the fluorescent brightness is to restrict the intermolecular rotation of fluorophores by rigidifying the conjugated backbone structure and enhancing the molecular coplanarity. Generally, classic polymethine fluorophores are composed of the flexible tunable long π-conjugation chain. Conformationally restraining the polymethine chain can improve their fluorescence quantum yields. For example, Schnermann groups reported a cascade cyclization strategy that attaches a tetracyclic ring system to the polymethine cyanine bridge.^36^ And this conformational restrain provides the constrained cyanine with more than four-fold enhancement of fluorescent brightness compared to similar non-constrained ones. Inspired by it, the dyes CX-2 and CX-3 were rationally designed by covalently conjugating two xanthene units with partially structural limited methine moieties for brighter fluorescence (Fig. 2D).^38^ And the dye 5H5 is also a partially rigidified polymethine fluorophore with outstanding NIR-II optical performance.^35^ In addition to partially restrained terminal methine moieties, five-membered or six-membered rings were introduced in the center of polymethine chain structure to prevent the conformational transition to improve the brightness, such as FD-1080,^26^ HCs,^39^ and Flav7,^24^*etc.*

### 2.3 Enhance NIR-II fluorophore stability

The chemostability and photostability of fluorophores in a complex microenvironment is also regarded as a matter worth profoundly considering in the development of NIR-II polymethine fluorophores which is of great importance for high-quality and high-contrast bioimaging. Unfortunately, the chemostability of NIR polymethine fluorophores is inherently poor on account of the small HOMO–LUMO gap. A too low LUMO orbital is easy to be attacked by nucleophiles, such as ubiquitous intracellular cysteine, homocysteine, glutathione, and so on. On the contrary, a too high HOMO orbital is susceptible to oxidation or electrophilic attack.^57^ And the majority of long-wavelength chromophores are notoriously unstable when exposed to light irradiation. For example, polymethine cyanine dyes, like ICG and Cy7, can photo-induce the generation of reactive oxygen species (ROS) *in situ* to decompose the polymethine chains which is the leading cause of fluorophore photobleaching.^58,59^ Hence, avoiding the reactive species attack as far as possible may be beneficial for the improvement of chemostability. For example, introducing large steric hindrance substitutes or electron-withdrawing groups into polymethine dyes is an available approach to prevent small-molecular fluorophores from attacking by various reactive species. Moreover, combining the fluorophores with protein to present a stable protein-fluorophore complex not only can improve the fluorescence quantum yield, but also enhance the NIR-II fluorophore stability. In addition, intensive efforts should be made to largely maximize the rigidity and effectively reduce the isomerization behavior for more stable fluorophores. Therefore, there is no doubt that the performance of polymethine fluorophores can be remarkably strengthened by improving the rigidity of conjugated backbone structures. Considering the rigid of xanthene structure, CX-2 and CX-3 were synthesized by covalently linking two xanthene units with an odd number of methine moieties.^38^ They are partially rigidified fluorophores and stable in aqueous solution due to partial restriction of the polymethine chain. Similarly, 5H5 is also a partially rigidified polymethine cyanine dye with excellent optical properties for NIR-II fluorescence bioimaging.^35^ Otherwise, the asymmetric Rh1029 fluorophore composed of xanthene and benzo[*cd*]indolium moieties also performed very well in NIR-II bioimaging.^33^

### 2.4 The strategies of increasing water solubility

The water solubility of fluorophores is also an important factor for biological applications in the biological environment. So far, there are two commonly used strategies to increase the water solubility of NIR-II polymethine fluorophores: one is to embed hydrophilic groups on the structure of fluorophores; the other is to package hydrophobic fluorophores with micelle or other carriers.^12,60^ For example, the frequently-used sulfonic acid groups were chemically modified on the molecular structure of polymethine fluorophores to improve their water solubility, such as FD-1080,^26^ LZ-1105,^34^ and FINR1072,^36^*etc.* PEGylation is also an effective strategy to improve water solubility and biocompatibility functionality.^61^ 5H5 with a terminal alkyne group was covalently modified with cRGD-PEG_8_*via* Cu(i)-catalyzed azide–alkyne click reaction for improving tumor targeted ability and water solubility.^35^ Additionally, hydrophobic fluorophores can also be imparted water solubility *via* a non-covalent hydrophilic encapsulation method. Polyethylene glycol conjugated phospholipids (DSPE-mPEG) is one of the most commonly used hydrophilic encapsulation materials which shows enormous potential for clinical biomedical application. The hydrophobic fluorophores were changed into hydrophilic nanoparticles for further biological imaging applications through the nano-precipitation method, such as CXs,^38^ BTC1070,^32^ Flav7,^24^ Rh1029,^33^ IR1061,^23^ and HCs,^39^*etc.*

## 3 NIR-II bioimaging applications

NIR-II fluorescent imaging allows detailed imaging of diverse biological processes with deep tissue penetration depth, high spatial and temporal resolution, and high signal-to-noise ratio benefiting from the longer wavelength with negligible autofluorescence and tissue scattering. NIR-II small-molecular polymethine fluorophores have various intrinsically inherent advantages, such as clear molecular structure, good biocompatibility, superior metabolic properties, and minimal long-term toxicity, which exhibit great potential for NIR-II bioimaging applications.^62^ The past decade has witnessed tremendous progress in the development of NIR-II polymethine cyanine dyes for versatile bioimaging applications. Herein, we introduce some representative application examples of polymethine-based NIR-II fluorescent imaging research, such as tumor targeted imaging, dynamic vessel imaging, and intravital multicolour imaging.

### 3.1 NIR-II tumor and dynamic bioimaging

**Fig. 4:**
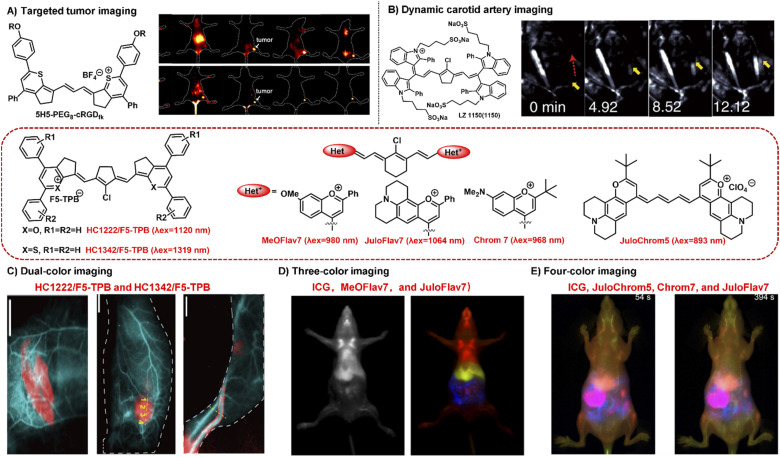
Typical NIR-II polymethine fluorophores for dynamic and multiplexed imaging. (A) The hydrophobic fluorophore 5H5 was transformed into 5H5-PEG_8_-cRGDfk through PEGylation which was used for *in vivo* NIR-II or NIR-IIa tumor targeted imaging. Reproduced with permission from ref. 35. Copyright 2019 American Chemical Society. (B) The water-soluble fluorophore LZ1105 was employed for dynamic tracking and imaging of the carotid artery, and the dynamic process of thrombolysis after injection of thrombolytics was distinctly visualized (yellow arrows). Reproduced with permission from ref. 34. Copyright 2020, Springer Nature. (C) HC1222/F5-TPB (*λ*_ex_ = 1120 nm) and HC1342/F5-TPB (*λ*_ex_ = 1319 nm) were employed to perform the dual-color imaging in living mice. Reproduced with permission from ref. 39. Copyright 2022 Wiley-VCH. (D) MeOFlav7 (*λ*_ex_ = 980) and JuloFlav7 (*λ*_ex_ = 1064 nm) combining with ICG (*λ*_ex_ = 785) were used for three-colour real time *in vivo* imaging. Reproduced with permission from ref. 25. Copyright 2020, Springer Nature. (E) Combining ICG (*λ*_ex_ = 785 nm), JuloChrom5 (*λ*_ex_ = 892 nm), Chrom7 (*λ*_ex_ = 968 nm), and JuloFlav7 (*λ*_ex_ = 1065 nm) can realize the four-color video-rate imaging in mice. Reproduced with permission from ref. 68. Copyright 2022 American Chemical Society.

NIR-II bioimaging technique can clearly discriminate the anatomical structures (*e.g.*, tumor, vasculature, lymph nodes, and skeleton) and monitor the dynamic physiological processes (*e.g.*, blood perfusion, ischemia, and arterial thrombosis) owing to its high temporal and high spatial resolution and deep tissue penetration.^58,63^ NIR-II polymethine fluorophores have been successfully applied in tumor imaging *in vivo*, showing great penetration depth, outstanding spatial resolution, and high signal-to-noise ratio. For example, the polymethine thiopyrylium salt 5H5 has both absorption and emission in the NIR-II window with a maximum absorption at 1069 nm and maximum emission at 1125 nm.^35^ To improve the tumor targeting ability and water solubility, the small-molecular fluorophore 5H5 with terminal alkyne function was covalently conjugated with an integrin-targeted peptide c(RGD)fk *via* copper-mediated azide–alkyne cycloaddition. *In vivo* NIR-II or NIR-IIa targeted imaging of human U87MG glioma, the highest tumor contrast was achieved at 8 h with a tumor tissue/nontumor tissue (T/NT) value of 4.0 upon the 808 nm laser irradiation, whereas the T/NT value was increased up to 6.8 under the 1064 nm excitation (Fig. 4A). This work not only provides a promising polymethine fluorophore for both NIR-II and NIR-IIa targeted bioimaging, but also convincingly demonstrates the prominent advantages of NIR-II excitation over NIR-I *in vivo* deep tissue bioimaging.

Real-time dynamic imaging and tracking the physiological process of hemodynamics dysfunction in blood flow is highly significant for preclinical biomedical research.^61,64^ The prerequisite for NIR-II fluorophores for the real-time dynamic monitoring of vascular diseases is that they should have enough long circulation time. LZ1105 containing four sulfonate groups have favorable NIR-II optical performance with the absorption and emission beyond 1000 nm.^34^ Specially, LZ1105 has long-term blood circulation with a half-life of 3.2 h which exhibits great potential for continuous real-time dynamic tracking of blood flow processes. In the carotid artery thrombosis mouse model, the dynamic processes of thrombolysis and blood flow recovery after treatment with recombinant tissue plasminogen activator (rt-PA) were clearly visualized by means of the NIR-IIa fluorescence of LZ1105 (Fig. 4B). Through the NIR-II fluorescence for *in vivo* real-time dynamic imaging and tracking, the potentially fatal massive hemorrhage possibly could be effectively prevented by regulating the injection of rt-PA. This work not only provides an early diagnosis strategy for fatal massive hemorrhage, but gives a research method for the pathogenesis study of vasculature-related diseases at small animal level. In addition, FD-1080 has also been successfully applied in hindlimb vasculature, brain vessel bioimaging, and dynamic imaging of respiratory craniocaudal motion with deep tissue penetration and high resolution benefiting from its excellent NIR-II optical performance, good aqueous solubility, and high stability.^26^

### 3.2 Multiplexed imaging

Multiplexed fluorescence imaging techniques have been developed as a powerful technique to explore the complexity of the biological systems, which benefit from the ability to clearly distinguish various cellular components or allow the selective simultaneous visualization of multiple biological species in one biological sample.^65–67^ To enable real-time multiplexed imaging *in vivo*, it is necessary to meet the following three requirements: (1) detection in the NIR-II window with high spatial resolution; (2) establishing multiple orthogonal excitations and/or emission detection wavelengths without the problem of crosstalk between different color channel; (3) rapidly detecting each channel on the millisecond timescale.^25^ NIR-II polymethine fluorophores have high molar extinction coefficients, intense fluorescent brightness, and particularly flexible wavelength tunability, which exhibits tremendous potential for multiplexed fluorescence imaging. For example, HC1222/F5-TPB and HC1342/F5-TPB have orthogonal excitation wavelengths (1120 and 1319 nm) and emission detection channels (channel 1: 1200–1300 nm; channel 2: 1450–1700 nm) which is beneficial for multiplexed imaging.^39^ The standard dual-color imaging of the circulatory system and metabolic organs was carried out in living mice through successively performing oral or intravenous injection of HC1222/F5-TPB nanoparticles and intravenous administration of HC1342/F5-TPB nanoparticles (Fig. 4C). The fine structures of the circulatory system, lymphatic structures, and tumor were distinctly resolved and distinguished under pseudo colors with a high signal-to-noise ratio. This work successfully extends multiplexed optical imaging ability, showing heptamethine cyanines as a promising candidate for deep tissue multicolour bioimaging.

In addition, Sletten's group also successfully developed two fluorophores MeOFlav7 and JuloFlav7 by changing the substituents of Flav7, which can well match the 980 and 1064 nm laser irradiation, respectively. By combining with the clinically approved ICG excitation at 785 nm as the third channel, they finally achieved three-colour real-time *in vivo* imaging with high temporal and spatial resolutions (Fig. 4D).^25^ This technology provides a new paradigm to imagine and track the orthogonal function in mammals, even in awake animals. Subsequently, combining these four spectrally distinct fluorophores ICG (*λ*_ex_ = 785 nm), JuloChrom5 (*λ*_ex_ = 892 nm), Chrom7 (*λ*_ex_ = 968 nm), and JuloFlav7 (*λ*_ex_ = 1065 nm), Sletten's group successfully realizes the four-color video-rate imaging in mice which is the first time to be reported (Fig. 4E).^68^

## 4 NIR-II biosensing imaging applications

Fluorescent biosensing in the NIR-II window can provide us an avenue to visualize or track biologically important analytes in deep tissue with outstanding spatial/temporal resolution. With the favor of NIR-II fluorescent probes, the biological and pathological processes in deep tissue can be clearly visualized and elucidated. NIR-II polymethine fluorophores have been widely employed to develop biosensing probes for diverse analytes. The commonly used sensing mechanisms in the development of molecular sensing mainly include photoinduced electron transfer (PET), excited-state intramolecular charge transfer, and Förster resonance energy transfer (FRET), *etc*.^69^ Of course, some other sensing mechanisms can also be employed to develop chemosensors, such as the conjugation system regulation. So far, there are two kinds of polymethine-based NIR-II fluorescent probes for biosensing, namely off–on probes and ratiometric probes, which have been developed and employed for biosensing imaging applications.

### 4.1 Off–on probes for biosensing

**Fig. 5:**
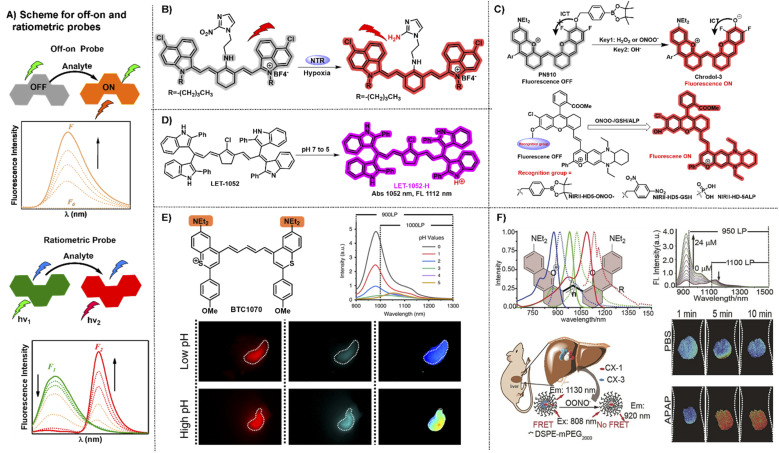
Representative NIR-II off–on and ratiometric probes based on polymethine fluorophores. (A) Schematic illustration of the off–on and ratiometric probe sensing mechanism and corresponding fluorescence spectra change pattern. (B) Catalyzed mechanism of the probe IR1048-MZ activated by NTR. Reproduced with permission from ref. 22. Copyright 2018 Wiley-VCH. (C) Responsive mechanism of PN910 and NIR-II-HD5-analyte (ONOO^−^, GSH, and ALP) activated by the corresponding analytes of interest based on ICT mechanism. Reproduced with permission from ref. 72 and 73. Copyright Wiley-VCH. (D) The viscosity-activatable probe LET-1052 was used to evaluate the therapeutic efficacy. Reproduced with permission from ref. 75. Copyright 2022 Wiley-VCH. (E) Chemical structure of BTC1070 and its fluorescence spectra at varied pH values and fluorescence imaging of gastric pH in mice stomach. Reproduced with permission from ref. 32. Copyright 2020, Springer Nature. (F) Top left: chemical structure and spectra of CX derivatives; bottom left: the schematic illustration of the detection mechanism of PN1100; top right: fluorescence spectra at 920 nm and 1130 nm; bottom right: ratiometric fluorescence images of the mice livers. Reproduced with permission from ref. 38. Copyright Wiley-VCH.

“Always-on” NIR-II fluorophores for bioimaging inevitably produce extra background signals through accumulation and retention at the non-target area. In contrast, activatable NIR-II probes can transform their fluorescence emission wavelength or intensity in response to specific physiological parameters or clinically relevant analytes, exhibiting a higher signal-to-noise ratio, higher specificity, and lower limit of detection compared to “always-on” fluorophores (Fig. 5A).^70,71^ NIR-II polymethine fluorophores were chemically tailored to construct off–on probes for biosensing due to their merits of flexible modification and wavelength tunability. A nitro group, exerted as a common fluorescence quencher, is generally adopted to develop activatable fluorescent probes. For example, Cai groups developed a hypoxia activatable NIR-II fluorescent probe IR1048-MZ through covalently conjugating the nitroreductase (NTR) enzyme-responsive nitro imidazole group with the commercially available NIR-II fluorophore IR-1048 (Fig. 5B).^22^ The fluorescence of IR-1048 is quenched by the nitro-imidazole moiety and the fluorescence emission intensity at 1046 nm displayed 106.9-fold enhancement when the nitro imidazole group was reduced from nitro to amino by NTR enzyme. This probe IR1048-MZ realized high-contrast tumor visualization with a clear boundary by NIR-II fluorescence imaging, but also afforded deep-tissue penetration at the centimeter level by 3D photoacoustic imaging.

ICT is an effective and popular sensing mechanism which is usually exerted in off–on probes. For example, Zhang and Lei groups developed a NIR-II fluorescent probe PN910 toward H_2_O_2_ and ONOO^−^ at pH over 7.4 with high selectivity and deep tissue penetration depth *in vivo* (Fig. 5C).^72^ In the cystitis and colitis mouse models, PN910 shows a remarkable fluorescence signal enhancement compared with the control. Otherwise, it was further proved that PN910 achieved reliable detection results instead of false-positive results through the biochemical analysis experiments. Therefore, this work provides a simple but effective chemical tool to monitor H_2_O_2_ and ONOO^−^ related to various diseases in an alkaline environment with high specificity and accuracy. To further improve the versatility of NIR-II activatable small-molecule probes, Yuan groups designed and developed a versatile platform based on ICT mechanism aiming at creating NIR-II activatable probes for analytes of interest, like ROS, thiols, and enzymes (Fig. 5D).^73^ This work not only provides multiple NIR-II activatable probes for different diseases in mouse models, but also gives a new paradigm to develop a widely adopted platform for more analytes testing in this field of biosensing in the NIR-II window. Similarly, Zhang and Yuan groups also developed multiple NIR-II platforms for the development of activatable fluorescence probes and synthesized three activatable probes NIRII-RT (NIRII-RT-pH, NIRII-RT-ATP, and NIRII-RT-Hg) for pH, adenosine triphosphate (ATP), and metal-ion detection to verify the universality of these NIR-II platforms.^74^

The real-time evaluation of tumor therapeutic efficacy during the treatment process is extremely important for successful tumor treatment. To achieve this goal, Lin *et al.* designed and developed a pH or viscosity activatable fluorescent probe LET-1052 for acidic tumor microenvironment (TME) triggered NIR-II PTT, along with viscosity triggered the assessment of therapeutic efficacy by itself.^75^ In acidic TME, the maximal absorption of LET-1052 at 1052 nm displays a 3.4-fold enhancement from pH 7.4 to 5.0 on account of the protonation on the nitrogen atoms and conjugated π-electron rearrangement, which activates NIR-II PTT and tumor-specific fluorescence imaging upon 1064 nm light irradiation. Afterward, PTT-induced cellular death brings about the elevated intracellular viscosity, along with the emerging NIR-I fluorescence signal for real-time evaluation of therapeutic efficacy due to the restricted intramolecular rotation of LET-1052. This work provides an advanced therapeutic modality with instant self-evaluation functionality for modern precise personalized medicine.

### 4.2 Ratiometric fluorescent imaging

Compared with off–on probes, ratiometric fluorescent probes with a built-in self-calibration for analytes of interest, enable the acquisition of quantitative or semiquantitative detection and bioimaging with high contrast and sensitivity.^76,77^ ICT is a frequently used sensing mechanism to develop NIR-II ratiometric fluorescent probes. For example, BTC1070 decorated with two amino groups possesses the absorption/emission beyond 1000 nm and has been calculated p*K*_a_ values at 0.29 and 3.81.^32^ BTC1070 exhibits a ratiometric fluorescence emission signal transformation from 1065 nm (BTC1070) to 980 nm (protonated BTC1070, namely BTC1070H_2_^2+^) between pH 1–4 which is attributed to the intramolecular charge transfer process of the amino groups switching from on to off (Fig. 5E). This work provides a reliable accurate probe to evaluate gastric pH at a 4 mm depth reference to the standard pH electrode method and successfully demonstrates the enormous potential of pentamethine cyanine fluorophores for various NIR-II biosensing applications.

Constructing the FRET system is also an alternative and powerful avenue to obtain a ratiometric fluorescence probe. Based on the FRET strategy, the fluorescence probe PN1100 composed of CX-1 and CX-3 dyes in micelles can be exerted as a ratiometric nanoprobe to monitor drug-induced hepatotoxicity by responding to corresponding marker analyte OONO^−^ (ref. 38) (Fig. 5F). The wavelength of the CX dyes can be effectively regulated with a reliable ratiometric fluorescence signal change from 1130 nm (CX-3) to 920 nm (CX-1) in response to the corresponding analyte. The *in vivo* ratiometric imaging demonstrates that the NIR-II fluorescence sensor PN1100 has the ability to precisely detect ONOO^−^ in the drug-induced hepatotoxicity mouse model, showing great potential for *in vivo* bio-detection applications. To realize adjustable pH detection in complex and variable tumor microenvironment, pH transition point (pHt) adjustable sensors, namely pTAS, were established by encapsulating the donor aza-BODIPY (NAB) with NIR-II emission and pH sensitive polymethine based pre-acceptor (NRh) to form the FRET system through simply regulating the component ratio.^78^ Combining the pH response regions of pTAS, a two-fold widened pH detection range (6.11–7.22) is obtained compared to the pHt settled sensor (6.38–6.94). With the help of the adjustable pHt, the real-time dynamic variation processes of tumor pH *in vivo* could be quickly captured through dual-channel ratiometric bioimaging in the NIR-II window which displays an ideal coefficient of variation under 1% reference to the standard pH meter.

## 5 Summary and outlook

NIR-II small-molecular polymethine fluorophores possess multiple intrinsically inherent advantages, such as clear molecular structure, good biocompatibility, rapid pharmacokinetic metabolism, minimal long-term toxicity, and flexible synthesis and modification. Delightedly, some NIR-II small-molecular polymethine fluorophores have been successfully developed and employed for various bioimaging and biosensing applications with outstanding resolution and high sensitivity. However, most of the available NIR-II polymethine fluorophores or probes have not been profoundly explored and primarily remained at the level of preclinical studies due to their self-limitation. To obtain deep penetration and high-fidelity imaging results for future clinical applications, some pending challenges are required to be addressed.

As discussed above, an ideal polymethine fluorophore should have high enough fluorescent brightness in the NIR-II spectral region with favorable stability and biocompatibility in a biological system. In the past decade, various strategies have been employed to push the absorption/emission peaks of polymethine fluorophores to more bathochromic shifts, mainly including lengthening the polymethine chain, modifying the heterocycle, and constructing stable fluorophore-protein J-aggregates, *etc.* Recent studies shows that imaging in the longer NIR-IIa/IIb regions (NIR-IIa, 1300–1400 nm; NIR-IIb, 1500–1700 nm) can obtain higher-quality images with almost “zero” background due to their minimized photon scattering, negligible autofluorescence, and deeper tissue penetration.^58^ However, the number of polymethine fluorophores with the absorption and/or emission wavelengths beyond 1200 nm remains rare. At the same time, the “cyanine limit” problem can never be ignored since fluorophores with the flexible long-wavelength polymethine chain usually encounter this dilemma. Crossing the “cyanine limit” generally leads to broader charge-transfer type absorption bands and lower fluorescence brightness of polymethine fluorophores which tremendously restricts their further biological imaging applications. One feasible manner to overcome this issue is not to lengthen the conjugation chain length, but rationally regulate and enhance the heterocyclic terminal unit, such as fluorophores Fav7 and its derivatives, C5, and 5H5. Conformational restraint and heterocycle exchange may also be exerted as alternative and feasible ways to obtain NIR-II emission while not crossing the cyanine limit, such as CX dyes, FNIR1072, HC dyes, and Flav7 analog dye. Therefore, it is futile to blindly chase the expansion of absorption or emission wavelengths in the NIR-IIa/IIb window. What even more important is that increasing wavelength to profoundly consider the fluorescent brightness in the biological aqueous environment for feasible imaging applications.

The fluorescent brightness of fluorophores in aqueous solutions directly determines the success or failure of bioimaging applications. Most polymethine fluorophores have enough long emission wavelength in the NIR-II window, but it is regrettable that they generally encounter the bottleneck of extremely low fluorescent brightness. In the development of pursuing more long-wavelength fluorophores in the NIR-II spectral region, it is challenging but valuable to reserve the fluorescent brightness of polymethine fluorophores in aqueous solutions. There are three common strategies to improve the fluorescent brightness, including introducing large steric hindrance, constructing a stable protein-fluorophore complex, and increasing the rigidity of polymethine skeleton structure. Besides, improving the stability and biocompatibility of polymethine fluorophores is also an unavoidable issue for successful application practice. Most of the NIR-II polymethine cyanine dyes in aqueous solution are not stable enough and vulnerable to reactive species which largely limits their further *in vivo* applications. This matter may be prevented by introducing large steric hindrance *via* covalent or non-covalent manners on the fluorophore skeleton. At the same time, the hindrance may also avoid the self-aggregation of fluorophores in aqueous solutions. Strengthening the rigidity of the conjugated backbone structures may also significantly improve the performance of polymethine fluorophores. In short, an ideal polymethine dye should not solely have a single outstanding advantage, but possess excellent comprehensive performance, especially high fluorescent brightness with a long wavelength in an aqueous solution.

In addition, more intensive efforts should also be devoted to the development of polymethine-based NIR-II multiple-detection probes for biosensing. Up to now, some NIR-II polymethine-based fluorescent probes have been successfully developed and used for biological detection, such as NIR-II HD 5 series, PN1100, PN910, and LET1052, *etc.* But it remains challenging to simultaneously track or even quantify multiple relevant analytes *in vivo* through one single probe. However, the biological microenvironment is a dynamic and complex network. Exploring the physiological processes or distinctly elucidating pathogenesis and realizing the multiple factors sensed simultaneously in living systems is of great importance for modern biochemistry and biomedicine. Hence, future efforts should be devoted to developing NIR-II fluorescent probes for multi-analyte detection, especially those probes that can respond to different analytes with orthogonal fluorescent signals or multimodal signals.

Rationally combining NIR-II polymethine fluorophores with other optical imaging or advanced biotechnology tactics is challenging but intriguing to further improve the imaging performance and extend their *in vivo* bio-applications. For example, bioluminescence and chemiluminescence imaging, as autofluorescence-free optical imaging which do not require extrinsic light excitation, can remarkably improve the signal-to-noise ratio.^79^ Combining bioluminescence and chemiluminescence imaging with NIR-II polymethine fluorophores to bathochromically shift their emission wavelength reaching the NIR-II window is still a great challenge. To achieve this, it requires highly efficient energy transfer systems between the donor (chemiluminescence functional groups) and the acceptor (NIR-II polymethine fluorophores) which is envisioned to bring about significant improvement in the spatial resolution. Besides, exploring the NIR-II polymethine fluorophores based on the biorthogonal chemistry would extend theirs *in vivo* imaging applications. The labeling strategy based on metabolic engineering and bioorthogonal chemistry is an ideal artificial targeting and labeling technology without interfering with native biochemical processes. One straightforward method to introduce this labeling technology into NIR-II polymethine fluorophores is to modify them with bioorthogonal tags (*e.g.*, cyclooctynes, ketones, and tetrazine). Of course, combining the NIR-II polymethine fluorophores with tagged proteins also can significantly improve fluorescent labeling efficiency in biological applications.

## Author contributions

The manuscript was written through contributions of all authors. All authors have given approval to the final version of the manuscript.

## Conflicts of interest

There are no conflicts to declare.

## Supplementary Material
